# Lymphomas affecting the submandibular glands

**DOI:** 10.4317/medoral.26065

**Published:** 2023-07-10

**Authors:** Gabriela Ribeiro de Araújo, Ana Luísa Morais-Perdigão, Cinthia Verónica Bardález Lopez-de-Cáceres, Oslei Paes de Almeida, Pablo Agustin Vargas, Elena María José Roman-Tager, Bruno Augusto Benevenuto de Andrade, Ciro Dantas Soares, Carlos Cesar de Oliveira Ramos, Maíra Medeiros Pacheco de Andrade, Alexandre de Oliveira Sales, Hélder Antônio Rebelo Pontes, Ricardo Alves Mesquita, Felipe Paiva Fonseca

**Affiliations:** 1 Department of Oral Surgery and Pathology, School of Dentistry, Federal University of Minas Gerais, Belo Horizonte, Brazil; 2 Department of Oral Diagnosis, Piracicaba Dental School, University of Campinas, Piracicaba, Brazil; 3 Department of Oral Diagnosis and Pathology, School of Dentistry, Federal University of Rio de Janeiro, Rio de Janeiro, Brazil; 4 Getúlio Sales Diagnósticos, Natal, Brazil; 5 Service of Oral Pathology, João de Barros Barreto University Hospital, Federal University of Pará, Belém, Brazil

## Abstract

**Background:**

Lymphomas affecting the submandibular glands are very uncommon and few reports are currently available in the literature. Therefore, the aim of the current study is to describe the clinical and microscopic features of an original series of lymphomas affecting the submandibular glands.

**Material and Methods:**

The pathology files of two institutions were searched for lymphoma cases affecting the submandibular glands. The original hematoxylin and eosin, and immunohistochemical slides were revised by a pathologist for diagnosis confirmation following the revised 4th edition of the World Health Organization classification of tumours of haematopoietic and lymphoid tissues. Clinical data regarding age, sex, clinical manifestation, treatment, follow-up and status at last appointment were retrieved from the patients’ medical charts.

**Results:**

During the period investigated, 16 cases were included in the study. Females predominated (10:6) with a mean age of 57.8 years-old. Tumors usually presented as asymptomatic swellings. MALT lymphoma represented the most common subtype, followed by diffuse large B cell lymphoma and follicular lymphoma. Three patients died, one of them affected by plasmablastic lymphoma, one by DLBCL and one by MALT lymphoma.

**Conclusions:**

Low-grade B cell lymphomas predominate in the submandibular glands, but DLBCL and other subtypes may also be rarely diagnosed in this salivary gland.

** Key words:**Lymphoma, salivary gland, submandibular gland, malt lymphoma, follicular lymphoma, diffuse large b cell lymphoma.

## Introduction

Non-Hodgkin lymphoma (NHL) is one of the most common human cancers and is characterized by a wide range of clinical manifestations and a complex microscopic classification (1,2). Its incidence showed an increasing trend worldwide from 1990 to 2019 (3). However, the primary involvement of salivary glands is considered very uncommon.

Primary NHL of the salivary gland represent approximately 5% of all extra-nodal non-Hodgkin lymphomas and only 1.7% of all salivary gland tumors (4). Most NHL that occurs in the salivary glands are B-cell lymphomas (5-7) and the submandibular glands are the second most affected major salivary gland after the parotid glands. According to previous small series and individual case reports the most common subtypes affecting the submandibular glands are the marginal zone lymphoma of the mucosa-associated lymphoid tissue (MALT lymphoma), follicular lymphoma (FL) and diffuse large B-cells lymphoma (DLBCL) (7-10).

Given the lack of larger series available in the literature, the biological heterogeneity of this group of neoplasms, their unspecific clinical manifestations and because they are very rarely found in the submandibular glands, the diagnosis of lymphomas in this anatomical region remains a challenge, possibly leading to a late diagnosis and an inappropriate management, deserving to be further documented and investigated (10-12). Therefore, the aim of this study was to describe the clinicopathological features of a series of lymphomas affecting the submandibular glands.

## Material and Methods

- Ethics statement

This study was done with approval from the Ethics Committee of the Federal University of Minas Gerais, Brazil (CAAE: 58900722.1.0000.5149). All procedures were in accordance with the ethical standards of the committee for human experimentation (institutional and national) and with the Declaration of Helsinki of 1975, revised in 2008.

- Sample and data collection

All cases of lymphomas affecting the submandibular glands were obtained from pathology files of the Getúlio Sales Diagnostics Laboratory (Natal/Brazil) and the Immunohistochemistry Laboratory of the Piracicaba Dental School (University of Campinas) in a time period ranging from January 2003 and December 2019. Lymphoma cases that were known to originate from the neck or from the surrounding lymph nodes that extended and invaded the submandibular glands were not considered in this study. Original H&E-stained histological sections and immunohistochemistry slides were obtained for diagnosis confirmation, which followed the revised 4th edition of the World Health Organization classification for hematopoietic and lymphoid tissue tumors (13). Demographic and clinical data of the cases were obtained from the patient's pathology and/or medical records and comprised gender, age, clinical presentation, follow-up time, status at last follow-up and the possible manifestation of the disease elsewhere in the body.

- Data analysis

Descriptive analyses were carried out, with continuous variables expressed as mean, standard deviation (SD) and range, while categorical variables were expressed as absolute numbers and percentages. The SPSS software version 22.0 (IBM, Germany) was used for statistical analysis.

- Literature review

An electronic search was carried out in December 2022 using the database PubMed/MEDLINE to retrieve all previous reports of lymphomas affecting the submandibular glands that contained individual data available for consultation published from 2001, when the third edition of the WHO classification of hematopoietic and lymphoid tissue tumors was published. The search strategy comprised the following key-words: ("submandibular gland" OR "submandibular glands") AND ("lymphoma" OR “lymphomas”). A manual search on the articles’ references was also performed in order to expand the search. Lymphomas affecting other major salivary glands and those without diagnostic information including histologic and immunohistochemical data, were not included in this review.

## Results

**Figure 1:**
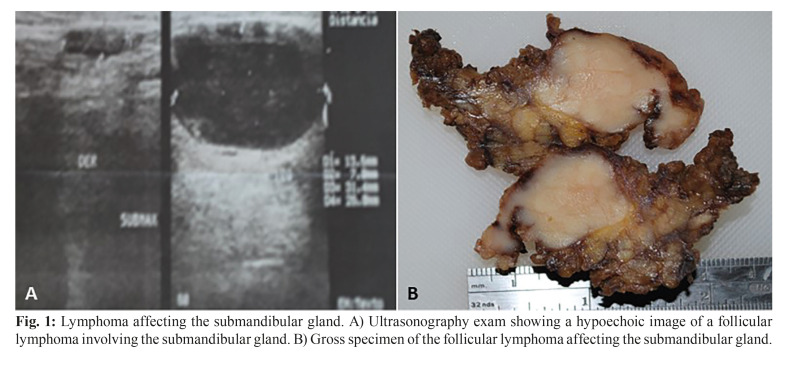
Lymphoma affecting the submandibular gland. A) Ultrasonography exam showing a hypoechoic image of a follicular lymphoma involving the submandibular gland. B) Gross specimen of the follicular lymphoma affecting the submandibular gland.

A total of 18 lymphoma cases affecting the submandibular glands were identified in the period investigated; however, the histological and/or immunohistochemical slides of two cases were not available for revision and, therefore, were excluded from the study. The clinicopathological data of the 16 cases included in this study are detailed described in Table 1. In summary, females predominated (10 cases:6 cases), and a mean age of 57.8 years-old was observed, ranging from 32 years-old to 87 years-old. Tumors presented as asymptomatic unilateral swellings in the submandibular region (Fig. 1). Regarding morphological distribution of the cases, MALT lymphoma represented the most frequent histological subtype (7 cases), followed by three cases of DLBCL NOS, 2 cases of follicular lymphomas, one plasmablastic lymphoma and one peripheral T cell lymphoma NOS. Two cases could not be further classified and received the diagnosis of small B cell lymphomas. Five cases were treated by surgery only, while four were treated with different chemotherapeutic schemes. Treatment data was not available for six cases. Follow-up data was available for 12 patients and ranged from 3 to 76 months, with a mean time of 26.4 months. Nine patients were alive free of disease, whereas three patients died (one affected by plasmablastic lymphoma, one by MALT lymphoma and one by DLBCL NOS).

**Table 1:**
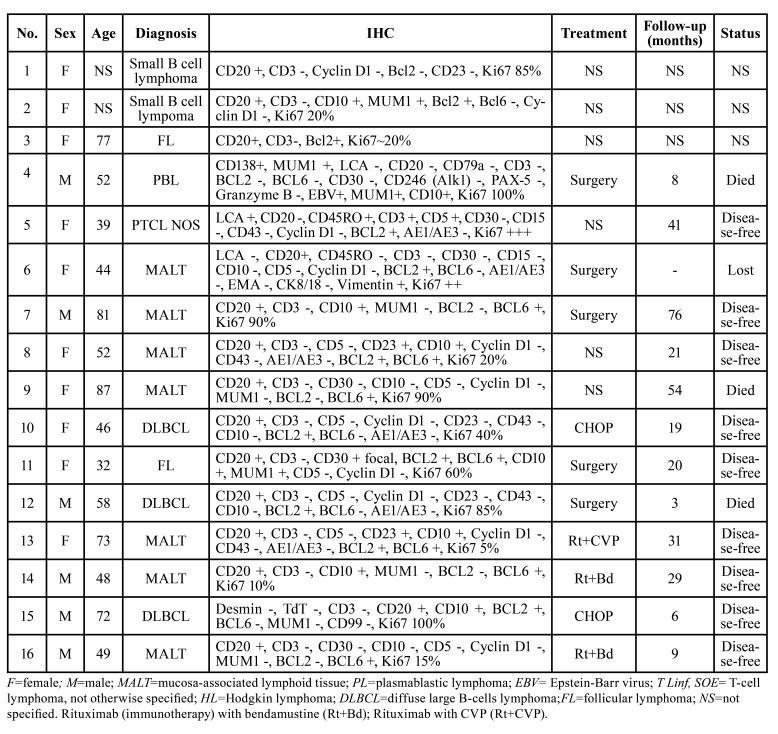
Clinicopathologic features of 16 lymphoma cases affecting the submandibular glands.

**Figure 2:**
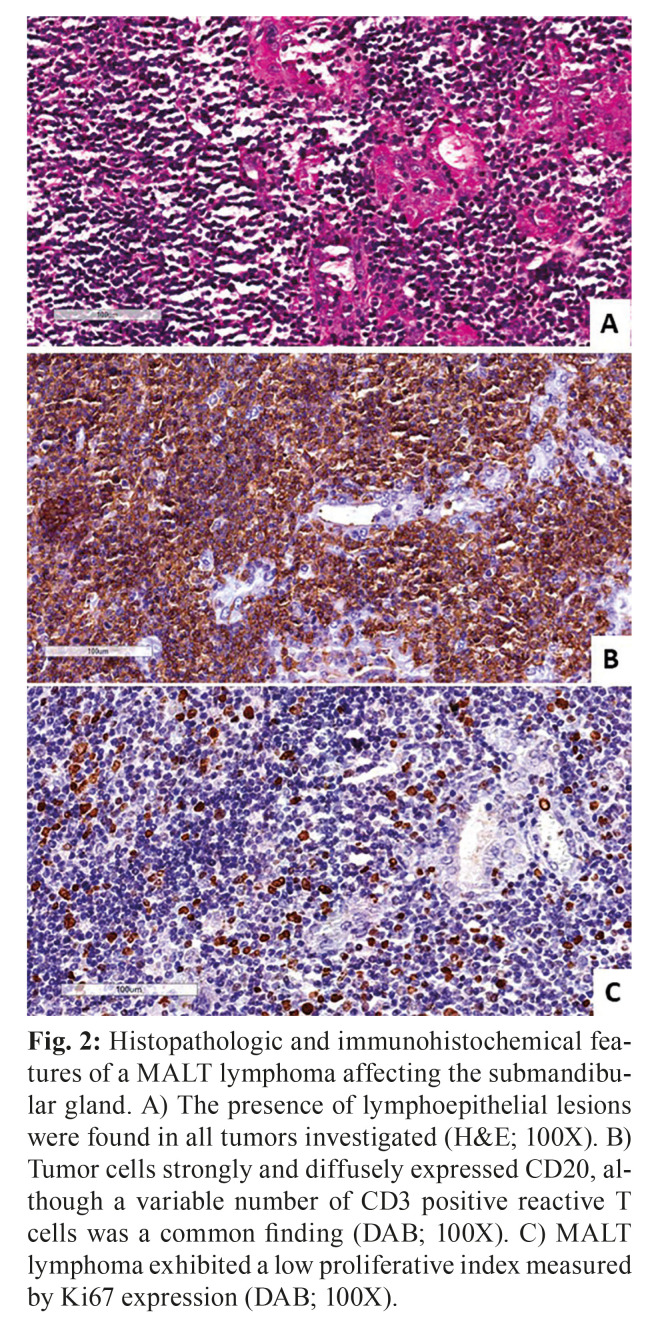
Histopathologic and immunohistochemical features of a MALT lymphoma affecting the submandibular gland. A) The presence of lymphoepithelial lesions were found in all tumors investigated (H&E; 100X). B) Tumor cells strongly and diffusely expressed CD20, although a variable number of CD3 positive reactive T cells was a common finding (DAB; 100X). C) MALT lymphoma exhibited a low proliferative index measured by Ki67 expression (DAB; 100X).

**Figure 3:**
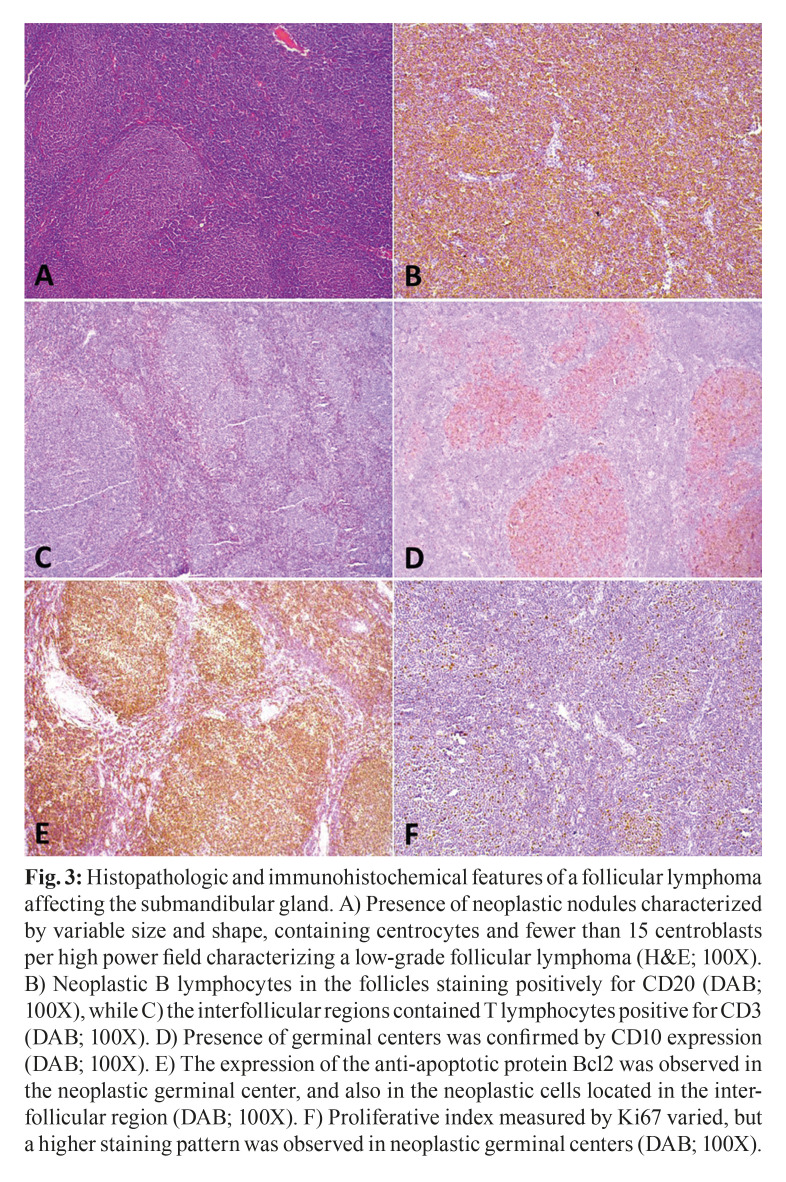
Histopathologic and immunohistochemical features of a follicular lymphoma affecting the submandibular gland. A) Presence of neoplastic nodules characterized by variable size and shape, containing centrocytes and fewer than 15 centroblasts per high power field characterizing a low-grade follicular lymphoma (H&E; 100X). B) Neoplastic B lymphocytes in the follicles staining positively for CD20 (DAB; 100X), while C) the interfollicular regions contained T lymphocytes positive for CD3 (DAB; 100X). D) Presence of germinal centers was confirmed by CD10 expression (DAB; 100X). E) The expression of the anti-apoptotic protein Bcl2 was observed in the neoplastic germinal center, and also in the neoplastic cells located in the interfollicular region (DAB; 100X). F) Proliferative index measured by Ki67 varied, but a higher staining pattern was observed in neoplastic germinal centers (DAB; 100X).

Microscopically, MALT lymphomas were characterized by the proliferation of small to medium-sized neoplastic B cell, with the frequent presence of plasma cells. Monocytoid B cells could be observed in all cases and lymphoepithelial lesions were present in all eight cases (Fig. 2). DLBCL NOS was microscopically heterogeneous and characterized be the presence of atypical large B cells with both centroblast and immunoblast features. The two cases of follicular lymphomas were both diagnosed as low-grade subtypes and revealed the presence or poorly defined neoplastic follicles proliferating in the glandular parenchyma (Fig. 3). Plasmablastic lymphoma was also comprised by large cells resembling plasmablasts that were positive for EBV detection. The only T-cell neoplasm of this sample represented a PTCL NOS case and exhibited a pleomorphic and heterogeneous microscopic aspect containing small and large cells.

In our literature review 21 articles reporting lymphomas in the submandibular glands could be retrieved, accounting for 30 cases. The clinicopathological data are detailed in Table 2 and references of this Table can be found in Supplement 1. Briefly, females predominated (20 females:10 males), with the patients’ age ranging from 24 to 75 years, and a mean age of 57.7 years. Most of the lesions presented as a firm, asymptomatic swelling, although discomfort on eating, fever and weight loss were also described. The most common treatment applied was surgery, with eight patients treated with surgery alone; three patients with surgery, chemotherapy and radiotherapy; five with chemotherapy and radiotherapy; six with chemotherapy only; one underwent radiotherapy only and one with surgery and radiotherapy. The information was not available for four patients. Twenty-two patients remained alive at their last follow-up, which ranged from 3 to 120 months. Microscopically, MALT lymphoma and Follicular lymphoma predominated, but DLBCL mantle cell lymphoma, extranodal NK/T-cell lymphoma, Burkitt’s lymphoma, and peripheral T-cell lymphoma, not otherwise specified were also described (Supplement 1).

**Table 2:**
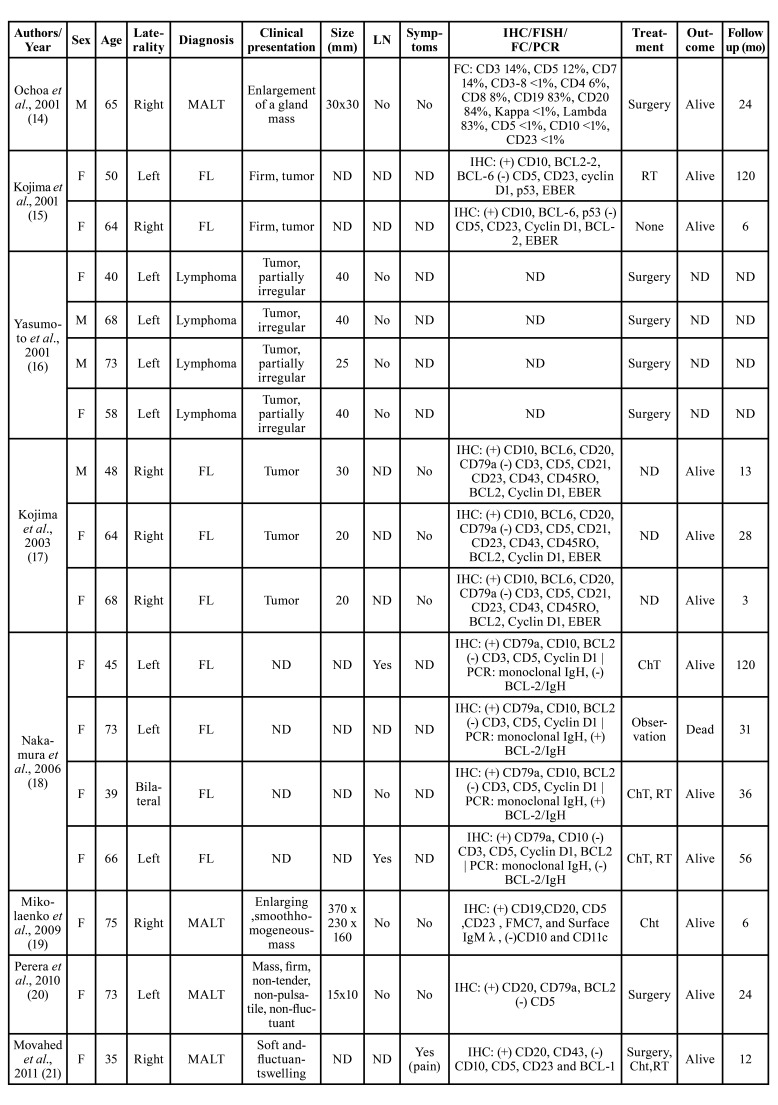
Clinical and microscopic features of lymphomas affecting the submandibular gland previously published in the literature.

**Table 2 cont.**
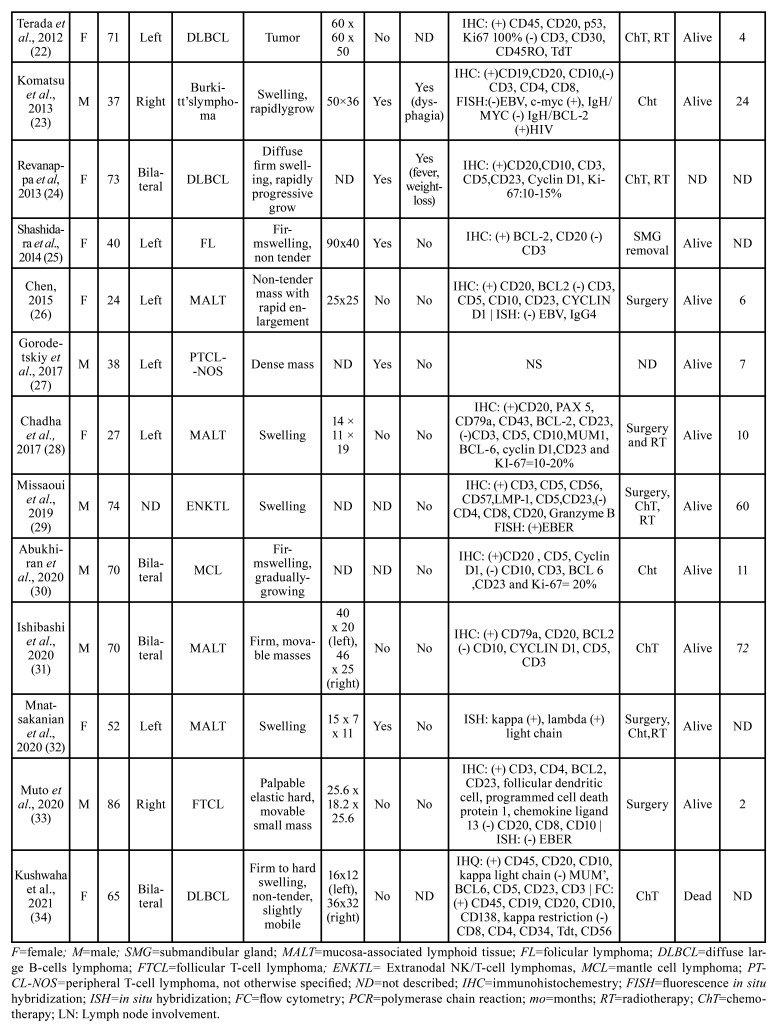
Clinical and microscopic features of lymphomas affecting the submandibular gland previously published in the literature.

## Discussion

Non-Hodgkin lymphomas represent a highly heterogeneous group of hematological malignancies that comprise a diverse number of subtypes in the WHO classification of lymphoid tissue tumors (13). They usually affect the lymph nodes, but extranodal manifestations are found in approximately 40% of the cases. The salivary glands is uncommonly affected and the parotid gland is the most involved, especially in patients with Sjögren syndrome (14), while very few cases were reported in the submandibular glands. In this series we demonstrated that MALT lymphoma, DLBCL NOS and follicular lymphoma were the most frequent entities in submandibular glands, similar to our previous study evaluating lymphomas in the sublingual glands (15).

Submandibular glands are located in the submandibular triangle, they have a superficial and a deep lobe separated by the mylohyoid muscle and their main excretory duct, Wharton’s duct, measures approximately 5 cm in length and 1.5 mm in diameter and drains inside the oral cavity (16). The submandibular gland is the second largest major salivary gland and its encapsulated tissue is considered a branched tubuloacinar gland composed of mucinous and serous acini, producing most saliva in the unstimulated state and different from parotid glands there is no intraglandular lymph nodes (16,17).

A large number of neoplasms can affect the submandibular glands, accounting for 7% to 11% of all salivary gland tumors and the clinical manifestation of these disorders are usually non-specific, manifesting as asymptomatic swellings (18). Approximately 30% to 54% of these neoplasms are malignant, more often carcinomas, especially adenoid cystic carcinoma, mucoepidermoid carcinoma and carcinoma ex-pleomorphic adenoma (18,19). However, mesenchymal (20) and lymphoid neoplasms (21) can also be found. Moreover, non-neoplastic diseases may also develop in these glands and sialolithiasis is one of the most common pathological conditions, frequently causing painful swellings (22). IgG4-related disease may also manifest in the submandibular glands and it should be considered in the differential diagnosis of lymphomas when this anatomic structure is evaluated for a lymphoid pathological process (23). The use of fine needle aspiration cytology to diagnose submandibular lesions is contributory, and it may aid in the management of the affected patients (19,24).

Only a few lymphoma cases affecting the submandibular glands have been described in the literature, most commonly manifesting as painless swellings in the cervical region. The current study represents one of the largest series described in the literature and we have also observed asymptomatic swellings as the most common clinical presentation of the disease. In accordance to our results, MALT and follicular lymphomas seems to be frequent subtypes in this region, with several cases being previously reported (25,26). However, the occurrence of higher-grade variants, especially DLBCL NOS has already been demonstrated (10,27). In our series, we identified two DLBCB NOS and one plasmablastic lymphoma. So far, we have not been able to find another case of plasmablastic lymphoma in these glands, demonstrating that pathologists must be aware for the occurrence of rare subtypes. Similarly, the presence of T cell lymphomas in submandibular glands is also very rare, with some single reports describing PTCL NOS (28) and FTH (29) lymphomas. In this study we included one case classified as PTCL NOS. The occurrence of these uncommon subtypes affecting the submandibular glands should also consider the possibility of tumor infiltration originating from surrounding lymph nodes that could not be demonstrated by histology analyses.

 The diagnosis of a lymphoma in the major glands demands from clinicians a special care for Sjögren syndrome, which increases the risk for the development of hematolymphoid neoplasms (14,25). In this scenario, MALT lymphoma is the most frequently diagnosed, but its distinction from reactive sialadenitis and follicular lymphomas may be often very difficult, demanding genetic evaluations. The presence of t(14;18)(q32;q21)/IGH-MALT1 is found in 15% of the cases and, together with the presence of immunoglobulin light chain restriction by immunohistochemistry or in situ hybridization, contributes to confirm the diagnosis of MALT lymphoma (30). Moreover, the use of imaging exams like ultrasound, not only collaborates with the diagnosis of the diseases, but it also represents a useful tool to confirm the involvement of the submandibular glands and to illustrate the limits of the disease, which is especially important considering the presence of various lymph node chains surrounding the gland (24).

As a consequence of a higher predominance of lower-grade B cell lymphomas in submandibular glands, the prognosis of the affected patients is usually favorable, with long survival rates. Depending on the microscopic subtype and also on tumor stage in the moment of diagnosis, treatment comprises surgery, radiotherapy and/or chemotherapy. Different schemes can be applied, but R-CHOP is usually the most used and significantly improved patients’ survival (10). In our series three patients passed away, two of them affected by high grade neoplasms, plasmablastic lymphoma and DLBCL NOS, and one affected by MALT lymphoma, who deceased due to another reason not related to the neoplasm.

In conclusion, we demonstrated that submandibular gland is more often affected by low-grade B cell neoplasms like MALT lymphoma and follicular lymphoma, which resembles the results obtained in our previous series of lymphomas affecting the sublingual glands. However, diagnosticians should be aware for high-grade subtypes affecting submandibular glands, especially DLBCL NOS.
